# Strange Freak of Nature

**Published:** 1869-10

**Authors:** 


### Strange Freak of Nature.

A child has been born of a shepherd's wife in Germany, that has a tumor at the lower portion of the spine, as large as a cocoanut. But what is strange about it is, that this tumor contains a living fœtus, whose well developed form can be distinctly felt through the thin walls of the tumor.

The Health Commissioner from Dirsharo, near where this child was born, refused to remove the tumor notwithstanding the urgent solicitations of the parents of child No. 1, for the reason that he believed child No. 2 would come to fruition.

He thought that no physician would be justified in destroying this marvelous being, but contrawise should protect it. So the matter rests, "waiting for something to turn up."

We will keep a lookout for the termination of this interesting case and inform our readers of the result in next BISTOURY.

---

—A wonderful submarine steamship has been examined and approved by the Parisian admiralty. It is a modification of the American monitor system, the deck and turret alone being above water ordinarily, but the vessel is capable of entire submersion, and can make an attack with submarine cannon and torpedoes, or sail under water during a storm.

—A man in New York City has for some time been advertising a process to remove freckles and tan. A lady in Ohio enclosed $1 for the information, and received the following: "By means of a sharp knife skin the parts affected."

—"Well, madam, how's your husband today?" "Why, doctor, he is no better." "Did you get the leeches?" "Yes; but he only took three of them raw—I had to fry the rest!"

—King William, of Prussia, is said to be a great success as an invalid. He drinks cognac to steady his nerves.

—The ladies do not furnish all the vain ones of the world. A Mr. Neal, of Tuftonboro, N. H., died last week from the effects of "Fowler's Solution," which he took for the purpose of "improving his complexion."

—Electricity on the railroads in France is taking the place of human watchfulness. On many lines there are contrivances where the passing of a train is automatically announced to neighboring stations.

